# Impact of Race-Free Glomerular Filtration Rate Estimations on CKD Prevalence in the US Military Health System: A Retrospective Cohort Study

**DOI:** 10.1016/j.xkme.2024.100861

**Published:** 2024-06-21

**Authors:** James D. Oliver, Robert Nee, Hava Marneweck, Amanda Banaag, Alain K. Koyama, Meda E. Pavkov, Tracey Pérez Koehlmoos

**Affiliations:** 1 Nephrology Service, Department of Medicine, Walter Reed National Military Medical Center, Bethesda, MD; 2 Henry M. Jackson Foundation for the Advancement of Military Medicine, Bethesda, MD; 3 Division of Diabetes Translation, Centers for Disease Control and Prevention, Atlanta, GA; 4 Department of Preventive Medicine and Biostatistics, Uniformed Services University of the Health Sciences, Bethesda, MD; 5 Department of Medicine, Uniformed Services University of the Health Sciences, Bethesda, MD

**Keywords:** Estimated glomerular filtration rate, chronic kidney disease, racial disparities in health care, Military Health System

## Abstract

**Rationale & Objective:**

The 2021 CKD-EPI removes Black race as a factor in calculating the estimated glomerular filtration rate (eGFR). We assessed its effect on CKD prevalence in the demographically-diverse US Military Health System.

**Study Design:**

A retrospective calculation of the eGFR from serum creatinine measured over 2016-2019 using both the 2009 and 2021 CKD-EPI equations.

**Setting & Population:**

Multicenter health care network with data from 1,502,607 adults in the complete case analysis and from 1,970,433 adults in an imputed race analysis.

**Predictors:**

Serum creatinine, age, sex, and race.

**Outcome:**

CKD stages 3-5, defined as the last eGFR persistently < 60 mL/min/1.73m^2^ for ≥90 days.

**Analytical Approach:**

The t test and Kruskal-Wallis test were used for continuous variables and Χ^2^ for categorical data.

**Results:**

The population in the complete case analysis had a median age of 40 years and was 18.8% Black race and 35.4% female. With the 2021 equation, the number of Black adults with CKD stages 3-5 increased by 58.1% from 4,147 to 6,556, a change in the crude prevalence from 1.47% to 2.32%. The number of non-Black adults with CKD stages 3-5 decreased by 30.4% from 27,596 to 19,213, a crude prevalence change from 2.26% to 1.58%. Similar results were seen with race imputation. Cumulatively, among adults with CKD stages 3-5 by at least one equation, 45.8% of Black adults were reclassified to more advanced stages of CKD and 44.0% of non-Black adults were reclassified to less severe stages across eGFR thresholds that could change clinical management.

**Limitations:**

Potential underestimation of CKD in individuals with only 1 measurement.

**Conclusions:**

Adoption of the 2021 CKD-EPI equation in the Military Health System reclassifies many Black adults into new CKD stages 3-5 or into more advanced CKD stages, with the opposite effect on non-Black adults. This may have an effect on CKD treatment and outcomes in ways that are yet unknown.

## Plain-Language Summary

Until recently, kidney function level was calculated from equations that adjusted the result if the individual was of Black race. Because this may contribute to racial disparities in kidney disease care, a new equation was developed in 2021 that excludes race as a factor. We assessed the possible effects of this equation using data from adults in the US Military Health System from 2016 to 2019. With the new equation, the number of Black adults classified with kidney disease increased while that of non-Black adults decreased. There were similar trends seen in the more severe levels of kidney disease, which could affect decisions in clinical care. These results emphasize the potential positive and negative outcomes to be monitored with the new equation.

For over 2 decades, the calculation of estimated glomerular filtration rate (eGFR) has included an adjustment factor for race because of data showing that Black adults on average have a higher serum creatinine than other racial or ethnic groups (American Indian or Alaska Native, Asian American or Pacific Islander, Hispanic, and White).^1^, ^2^, ^3^ In the 2009 equation developed by CKD-EPI, a separate coefficient for Black race resulted in eGFR for Black individuals being 16% higher than that of non-Black individuals with the same sex, age, and serum creatinine.^4^ In recent years, this practice has come under scrutiny as race is increasingly recognized as a social and nonbiological construct, and such an approach may contribute to disparities in CKD care in the United States.^5^ Consequently, in 2021, the National Kidney Foundation (NKF) and the American Society of Nephrology (ASN) Task Force on reassessing the inclusion of race in diagnosing kidney diseases^6^ issued a recommendation for the use of a new CKD-EPI creatinine-based eGFR equation that does not include race as a factor.^7^

However, the clinical implications of excluding race in estimations of kidney function are not yet known. The adjustment of eGFR has potential downstream effects on referral patterns for specialty care, in the application of clinical practice guidelines and selection of treatment options, and in eligibility for receiving or donating kidney allografts. In this study, we retrospectively quantified the population effects of the race-free 2021 CKD-EPI equation on the prevalence of CKD stages 3-5 in the US Military Health System (MHS). The MHS is an integrated health care network within the US Department of Defense, serving a diverse population of 9.6 million active and retired service members and their families. Universal health coverage in the MHS has been shown to mitigate disparities in health care for some conditions,^8^ although racial disparities in CKD are still seen.^9^

## Methods

Data for fiscal years 2016-2019 (October 1, 2015-September 30, 2019) were extracted from the MHS Data Repository (MDR) for adults (≥18 years) without a diagnosis of kidney failure (requiring long-term dialysis or transplant). The MDR houses health care information for MHS beneficiaries who receive care through TRICARE (the Department of Defense insurance product) at military treatment facilities or at civilian fee-for-service facilities.^10^ The TRICARE does not cover care delivered in either combat zones or the Veterans Affairs system.

The eGFR was calculated from serum creatinine obtained in outpatient and inpatient settings using both the 2009 and 2021 CKD-EPI equations. Age on the date of the laboratory test was used in the equations, whereas age on September 30, 2019 was used for summary tables in the results. A total of 1,970,995 adults had 7,121,880 measurements of serum creatinine. We removed 21,690 outliers (serum creatinine < 0.1 or > 25 mg/dL or eGFR > 200 mL/min/1.73m^2^), leaving a total of 1,970,433 adults and 7,100,190 values for analysis. Consistent with the NKF–KDIGO (National Kidney Foundation–Kidney Disease Outcomes Quality Initiative) guidelines,^11^ we defined CKD stages 3-5 as having the most recent eGFR values persistently <60 mL/min/1.73m^2^ for at least 90 days.

For those adults classified as having CKD, the 2 serum creatinine results that defined the eGFR stratum were separated by 90-360 days in 80.4% of adults and by 90-540 days in 94.6% (Table S1). The mean absolute difference (± standard deviation) in eGFR over all determinations was 5.3 ± 5.1 mL/min/1.72m^2^ with little change over the length of the determination period (Fig S1).

A total of 745,264 adults (37.8%) had only one creatinine measurement over the study period. Adults in this group with an eGFR of <60 mL/min/1.73m^2^ could not meet the diagnostic criteria for CKD but were used to estimate an upper bound on CKD prevalence.

In the MDR, biological sex is defined as female and male, while self-reported race is categorized as Black, White, Asian American and Pacific Islander, American Indian and Alaska Native, Other, and Unknown. (We use the term “Black” instead of “African American” in this study to be consistent with terminology used in the MDR and also that used by the NKF-ASN Task Force^6^ and the race-free CKD-EPI equation.^7^) Data on ethnicity in the MDR were sparse and were not used. A significant percentage of the population (467,826, 23.7%) did not have race reported and was coded as missing. Adults with missing races were overwhelmingly female (435,929, 93.2% of the missing race population) and not active-duty military (467,768, 99.9%). Our primary analysis was performed on the complete case of individuals with nonmissing race. A secondary analysis was performed with missing race assigned as Black or non-Black using the method of multiple imputation by fully conditional specification.^12^ Multiple imputation was based on the assumption that the missing race data were missing at random, given that the reason for the missing data was likely explained by other observed characteristics, such as sex and nonactive-duty status.^13^ We reported averages over 25 imputations in SAS using PROC MI, with 95% confidence intervals (CI) calculated by Rubin’s method.^14^

On entering the military, active-duty personnel undergo a screening process to exclude chronic diseases, such as CKD.^15^ Consequently, the risk of CKD is lower in the active-duty population but still carries specific implications for military readiness and deployment strength. As such, active-duty members were analyzed as a distinct cohort within the MHS.

Statistical significance (*P* < .05) was determined by t test for comparison of means, the Kruskal-Wallis test for comparison of medians, and Χ^2^ for categorical data. Non-zero cells with sizes < 11 individuals were not reported. The Uniformed Services University institutional review board exempted this study from review.

## Results

### Demographics

The population of the complete case analysis had a median age (interquartile range, IQR) of 40 (28-54) years and was 18.8% Black race, 35.4% female, and 50.2% active-duty military (Table 1). Henceforth, we will restrict discussion results comparing Black race with the combined category of non-Black race. Demographics for separate non-Black groups are shown in Tables S2a and S2b.

**Table 1:** MHS Demographics and CKD Stages 3-5 Number and Prevalence by 2009 Versus 2021 CKD-EPI Equations

All Adults			
Race	All	Black	Non-Black
No.	1,502,607	282,890	1,219,717
Proportion, %	100.0	18.8	81.2
Female, n	532,445	107,373	425,072
Female proportion, %	35.4	38.0	35.0
Age, (y) median (IQR)	40 (28-54)	41 (29-54)	39 (28-54)
Active-duty, n	754,111	150,858	603,253
Active-duty proportion, %	50.2	53.3	49.5
Median 2009 eGFR (IQR) mL/min/1.73m^2^	91 (75-106)	95 (79-113)	90 (74-104)
Median 2021 eGFR (IQR) mL/min/1.73m^2^	93 (77-108)	87 (72-103)	95 (78-109)
Median eGFR change (IQR), 2009 to 2021, mL/min/1.73m^2^	4 (2-5)	−8 (−11 to −6)	4 (3-5)
2009 CKD stages 3-5, n	31,743	4,147	27,596
2021 CKD stages 3-5, n	25,769	6,556	19,213
Change in CKD stages 3-5 n, %	−18.8	+ 58.1	−30.4
2009 CKD stages 3-5 crude prevalence, %	2.11	1.47	2.26
2021 CKD stages 3-5 crude prevalence, %	1.71	2.32	1.58
Change in crude prevalence, %	−0.40	+ 0.85	−0.68

Active-Duty Adults			
Race	All	Black	Non-Black
No.	754,111	150,858	603,253
Proportion, %	100.0	20.0	80.0
Female, n	175,897	52,391	123,506
Female proportion, %	23.3	34.7	20.5
Age, (y) median (IQR)	31.0 (25-39)	31 (25-39)	31 (25-39)
Median 2009 eGFR (IQR) mL/min/1.73m^2^	101 (87-116)	105 (89-123)	100 (86-114)
Median 2021 eGFR (IQR) mL/min/1.73m^2^	102 (88-117)	94 (80-110)	104 (90-118)
Median eGFR change (IQR), 2009 to 2021, mL/min/1.73m^2^	3 (0-4)	−11 (−13 to −9)	4 (3-4)
2009 CKD stages 3-5, n	838	216	622
2021 CKD stages 3-5, n	858	523	335
Change in CKD stages 3-5 n, %	+ 2.39	+ 142.1	−46.1
2009 CKD stages 3-5 crude prevalence, %	0.11	0.14	0.10
2021 CKD stages 3-5 crude prevalence, %	0.11	0.35	0.06
Change in crude prevalence, %	+ 0	+ 0.21	−0.04

### Changes in Numbers and Prevalence of CKD Stages 3-5

The median change in eGFR was –8 mL/min/1.73m^2^ in Black adults and +4 mL/min/1.73m^2^ in non-Black adults (Table 1). The number of Black adults with CKD stages 3-5 (eGFR of <60 mL/min/1.73m^2^) increased by 58.1% from 4,147 to 6,556, an increase in crude prevalence from 1.47% to 2.32 (+0.85 %). The number of non-Black adults with CKD stages 3-5 decreased by 30.4% from 27,596 to 19,213, a decline in crude prevalence from 2.26% to 1.58% (−0.68%). The overall population CKD stages 3-5 declined 18.8% from 31,743 to 25,769 with a decrease in crude prevalence from 2.11% to 1.71% (−0.40%).

The crude prevalence of CKD stages 3-5 in active-duty adults was lower at 0.11% and did not change with the 2021 equation (Table 1). However, the number of active-duty Black adults increased 142% from 216 to 523 with an increase in crude prevalence from 0.14% to 0.35% (+0.21%) while the number of active-duty non-Black adults decreased 46.1% from 622 to 335, with a decline in crude prevalence from 0.10% to 0.06% (−0.04%). Despite being only 20% of the active-duty population, the number of Black adults with CKD stages 3-5 exceeded the number of non-Black adults with the 2021 equation.

After imputation for missing race, prevalence of CKD stages 3-5 increased when compared with the complete case analysis in both Black and non-Black adults and for both equations (Table 2). However, the effects of using the 2021 equation with race imputation were similar to those for the complete case analysis: the number and prevalence of CKD stages 3-5 for Black adults changed +53.2% and +0.87% (when compared with +58.1% and +0.85% for complete cases) and for non-Black adults changed −28.3% and −0.75% (when compared with −30.4% and −0.68% for complete cases). Full results from race imputation are shown in Tables S2c-S2f.

**Table 2:** Comparison of Demographics and Changes in CKD Stages 3-5 in Complete Case Versus Imputed Race Analysis

	Black Adults		Non-Black Adults	
	Complete Case	Imputed Race	Complete Case	Imputed Race
No.	282,890	355,314 (354,064-356,563)	1,219,717	1,615,119 (1,613,870-1,616,369)
Proportion, %	18.8	18.0 (17.97-18.10)	81.2	82.0 (81.90-82.03)
Female proportion, %	38.0	49.6 (49.4-49.8)	35.0	51.0 (50.9-51.0)
Age (y), median (IQR)	41 (29-54)	44 (31-56)	39 (28-54)	40 (28-55)
Active-duty proportion, %	53.3	42.5 (42.3-42.6)	49.5	37.4 (37.4-37.4)
2009 CKD stages 3-5, n	4,147	5,828 (5,669-5,986)	27,596	43,126 (42,718-43,534)
2021 CKD stages 3-5, n	6,556	8,928 (8,735-9,121)	19,213	30,932 (30,586-31,279)
Change in CKD stages 3-5 n, %	+ 58.1	+ 53.2	−30.4	−28.3
2009 CKD stages 3-5 crude prevalence, %	1.47	1.64 (1.60-1.68)	2.26	2.67 (2.65-2.70)
2021 CKD stages 3-5 crude prevalence, %	2.32	2.51 (2.46-2.57)	1.58	1.92 (1.89-1.94)
Change in crude prevalence, %	+ 0.85	+0.87	–0.68	–0.75

*Note:* For n in imputed race results, 95% confidence intervals are shown in parentheses.

Abbreviations: CKD, chronic kidney disease; IQR, interquartile range.

We estimated an upper bound on CKD stages 3-5 prevalence by including those adults who had only a single creatinine measurement and an eGFR of <60 mL/min/1.73m^2^ (details in Tables S3a and S3b). From this estimate, crude CKD prevalence using the 2009 equation increased from 1.47% to 1.76% for Black adults, from 2.26% to 2.74% for non-Black adults, and from 2.11% to 2.56% overall. With the 2021 equation, the increases were 2.32% to 2.93% for Black adults, 1.58% to 1.87% for non-Black adults, and 1.71% to 2.07% overall.

### Reclassification to and From CKD Stages 3-5

A total of 2,409 Black adults were reclassified into CKD stages 3-5 by the 2021 equation, while 8,383 non-Black adults were reclassified out of CKD (Table 3). When compared with those reclassified out of CKD, adults reclassified into CKD were younger (median age [IQR] 60 [53-65] vs 71 [64-81] years; *P* < .001), more likely to be male (24.4% vs 20.4% female; *P* < .001), and more likely to be active-duty (12.7% vs 3.4%; *P* < .001).

**Table 3:** Comparison of Populations Reclassified Into and out of CKD Stages 3-5 by 2021 CKD-EPI Equation

	Black Adults Reclassified Into CKD With 2021 Equation	Non-Black Adults Reclassified out of CKD With 2021 Equation
n	2,409	8,383
Female, n (%)	587 (24.4)	1,708 (20.4)^a^
Age (y), median (IQR)	60 (53-65)	71 (64-81)^a^
Active-duty, n (%)	307 (12.7)	287 (3.42)^a^

Abbreviations: CKD, chronic kidney disease; IQR, interquartile range.

a *P* < .001 versus adults reclassified into CKD.

### Restratification Within Levels of CKD

**Figure 1:**
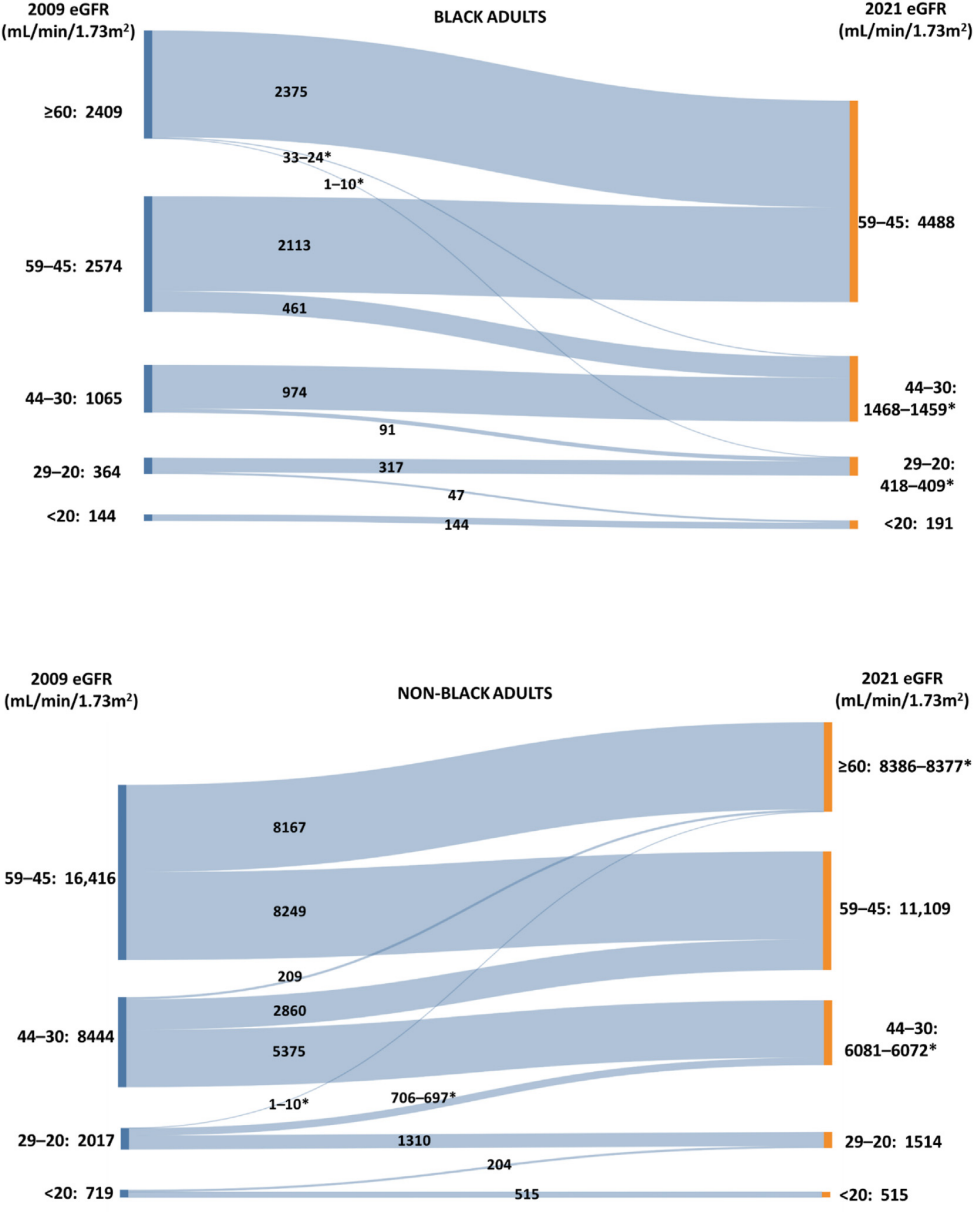
Sankey diagrams depicting redistribution of CKD populations among eGFR strata after application of 2021 CKD-EPI equation for Black (top) and non-Black (bottom) adults. The average of first and last eGFR over ≥90 days is used to determine strata. For clarity, the populations with an eGFR of ≥60 mL/min/1.73m^2^ in both equations (276,334 Black adults and 1,192,121 non-Black adults) are not shown. There were small numbers of Black adults with 2009 eGFR of ≥60 mL/min/1.73m^2^ who fell more than one stratum (n = 34) and non-Black adults who rose more than one stratum into 2021 eGFR ≥ 60 mL/min/1.73m^2^ (n = 210-219). These were adults with decreased eGFR but with persistence for ≥ 90 days only with the 2021 equation (Black adults) or only with the 2009 equation (non-Black adults). ∗Range of numbers reported to prevent explicit or implicit reporting of populations < 11 adults.

Table 4 and Fig 1 show the effect of changing equations at strata of eGFR important in clinical decision-making: medication adjustments^16^, ^17^, ^18^ or indications for nephrology referral or for prophylaxis against contrast-induced acute kidney injury^19^ at eGFR < 45 or < 30 mL/min/1.73m^2^ and active listing on kidney transplant waiting lists at eGFR < 20 mL/min/1.73m^2^.^20^ The 2021 equation increased the numbers of Black adults at higher stages of CKD and correspondingly decreased those for non-Black adults. The largest effect was observed at 59-45 mL/min/1.73m^2^: the number of Black adults in this range increased from 2,574 to 4,488, an increase of 74.4%, whereas the number of non-Black adults decreased from 16,416 to 11,109, a change of −32.3%. The number of Black adults with eGFR 44-30, 29-20, and < 20 mL/min/1.73m^2^ rose +37.8%, +12.4%, and +32.6%, respectively, while those for non-Black adults fell −28.1%, −24.9%, and −28.4%, respectively. Cumulatively, 3,008 Black adults and 12,147 non-Black adults were reclassified or restratified by the 2021 equation. After excluding adults with an eGFR of >60 mL/min/1.73m^2^ in both equations, this represented 45.8% (3,008/6,556) of Black adults, 44.0% (12,147/27,596) of non-Black adults, and 44.4% (15,155/34,152) of all adults classified with CKD stages 3-5 by at least one equation.

**Table 4:** Effect of 2021 CKD-EPI Equation on Reclassification Within Levels of CKD

eGFR Level (mL/min/1.73m^2^)	Clinical Significance	Race	n, 2009 CKD-EPI	n, 2021 CKD-EPI	Change From 2009 to 2021 Equation	% Change From 2009 to 2021 Equation
59-45	CKD diagnosis Exclusion as kidney donor	All adults	18,990	15,597	−3,393	−17.9
		Black adults	2,574	4,488	+1,914	+74.4
		Non-Black adults	16,416	11,109	−5,307	−32.3
		All active-duty adults	656	672	+16	+2.4
		Active-duty Black adults	154	431	+277	+180
		Active-duty non-Black adults	502	241	−261	−52.0
44-30	Medication dosing (eg, angiotensin receptor blockers)	All adults	9,509	7,543	−1,966	−20.7
		Black adults	1,065	1,468	+403	+37.8
		Non-Black adults	8,444	6,075	−2,369	−28.1
		All active-duty adults	132	134	+2	+1.5
		Active-duty Black adults	41	66	+25	+61.0
		Active-duty non-Black adults	91	68	−23	−25.3
29-20	Nephrology referral Medication dosing (eg, metformin) Risk of contrast-associated acute kidney injury	All adults	2,381	1,923	−458	−19.2
		Black adults	364	409	+45	+12.4
		Non-Black adults	2,017	1,514	−503	−24.9
		All active-duty adults	35	37	+2	+5.71
		Active-duty Black adults	17	21	+4	+23.5
		Active-duty non-Black adults	18	16	−2	−11.1
<20	Eligible for kidney transplant listing Medication dosing (eg, SGLT2i)	All adults	863	706	−157	−18.2
		Black adults	144	191	+47	+32.6
		Non-Black adults	719	515	−204	−28.4
		All active-duty adults	15	15	0	0
		Active-duty Black adults	1-10	1-10	a	a
		Active-duty non-Black adults	11-14	1-10	a	a

*Note:* The average of first and last eGFR over the most recent 90 days is used to determine level. Cell counts of < 11 are reported as 1-10. Some cells of active-duty adults are shown as a range of numbers to prevent explicit or implicit reporting of counts < 11.

Abbreviations: CKD, chronic kidney disease; CKD-EPI, chronic kidney disease epidemiology collaboration; eGFR, estimated glomerular filtration rate, SGLT2i: sodium-glucose cotransporter 2 inhibitor.

a Not calculated due to low cell numbers.

Within the active-duty population, similar patterns were seen: the number of individuals with an eGFR of 59-45, 44-30, and 29-20 mL/min/1.73m^2^ rose by +180%, +61.0%, and +23.5%, respectively, for Black adults and fell −52.0%, −25.3%, and −11.1%, respectively for non-Black adults. Numbers for active-duty adults with an eGFR of <20 mL/min/1.73m^2^ were too small to report.

## Discussion

Changing from the 2009 equation to the 2021 version in the MHS increased both the prevalence of CKD 3-5 and the prevalence of more advanced stages of CKD in Black adults, while having the opposite effect on non-Black adults. Although the prevalence of CKD stages 3-5 was much lower in the active-duty population, the relative magnitude of the shifts between Black and non-Black adults was amplified when compared with the overall study population. When we examined changes across specific strata of eGFR, we found that the 2021 equation could have consequences in clinical management for ∼45% of the population with CKD in the MHS.

We recently reported a crude prevalence of CKD stages 3-5 of 2.9% out of >3.3 million MHS beneficiaries in the fiscal year 2015, based on matching International Classification of Diseases, 9th Revision (ICD-9) codes.^21^ In this study, we calculated a crude CKD 3-5 prevalence using the 2009 and 2021 equations of 2.11% and 1.71% in the complete case analysis and 2.48% and 2.02% after race imputation. Thus, the overall prevalence of CKD in the MHS is similar between laboratory-based and diagnostic code-based criteria.

Several recent studies have analyzed effects of the 2021 CKD-EPI equation in other populations.^22^, ^23^, ^24^, ^25^, ^26^ Ghuman et al^24^ reported on a population of 170,941 adults at the University of Washington, while Diao et al^26^ projected the national impact by extrapolation from laboratory data of 44,360 adults in the National Health and Nutrition Examination Survey (NHANES). These studies reported median changes in the eGFR, which are comparable to the results reported here (−9.4 and +4.2 mL/min/1.73m^2^ for Black and non-Black adults, respectively at University of Washington and −10.5 and +3.27 to +3.95 mL/min/1.73m^2^ for Black and non-Black adults, respectively from NHANES). However, the relative changes in CKD prevalence for Black and non-Black adults are dissimilar among the studies: +23.4% and −20.8% (Ghuman et al^24^) and +10.9% and −19.7% (Diao et al^26^) when compared with +58.1% and −30.4% in this study. Some of the variation can be attributed to demographic differences in the populations studied but there are also likely methodological reasons. One important difference is that Diao et al^26^ included CKD stages 1-2 with albuminuria in their definition of CKD, while our study and that of Ghuman et al^24^ used only the eGFR criteria. Inclusion of albuminuria in CKD diagnosis would attenuate the proportion of reclassification based solely on eGFR changes. Another important difference is that, whereas we required chronicity over 90 days, both previous studies assigned CKD status based on single laboratory values, which is more likely to overestimate the true prevalence. Using single eGFR values from the 2021 equation to define CKD will increase overestimation when compared with the 2009 equation in Black adults while decreasing it in non-Black adults, although the relative magnitude of the effects is unclear.

Gregg et al^25^ analyzed data from the US Veterans Affairs health care system using a definition of CKD that, like the methods here, required an eGFR chronicity. In a population of 1.78 million veterans with CKD stages 3-4, they determined that the use of the 2021 CKD-EPI equation nationwide reclassified 66,190 Black adults with new CKD while reclassifying 289,242 non-Black adults out of CKD. Neither denominators for the racial subpopulations nor prevalence were reported in their study.

Our study findings suggest several possible consequences of wider promulgation of the 2021 CKD-EPI in the MHS. Referrals for CKD care for Black adults would be expected to increase, particularly for males who constituted 75.6% of the newly-reclassified CKD in our study. As Black adults in the United Sates have a substantially higher risk of kidney failure,^27^ this is a desirable effect. Ideally, this would result in more timely engagement with kidney specialists, performance of confirmatory testing such as creatinine-cystatin C eGFR when indicated,^7^ opportunities for patient education and shared decision-making, and earlier interventions to reduce morbidity and mortality. For those at highest risk earlier discussions about and preparations for dialysis or transplant is an important goal. However, there are potential drawbacks, such as fewer Black adults in the transplant donor pool, avoidance of diagnostic studies that require radiocontrast dye, and less prescribing of beneficial medications and at lower doses. For example, the 2022 American Diabetes Association (ADA)/NKF–KDIGO consensus report^18^ contains guideline-directed therapy recommendations for patients with type 2 diabetes mellitus and CKD, with eGFR thresholds for some medications. These included the use of sodium-glucose cotransporter 2 inhibitors at an eGFR of ≥20 mL/min/1.73m^2^, nonsteroidal mineralocorticoid receptor agonists at an eGFR of ≥25 mL/min/1.73m^2^ (in patients with albuminuria), and metformin at an eGFR of ≥30 mL/min/1.73m^2^. Black adults could be at risk for not receiving these therapies because of being restratified to more advanced stages of CKD, exacerbating the already-existing disparity in the use of new medications.^28^ Finally, there is likely to be an increase in Black adults who do not in fact have CKD undergoing potentially unnecessary additional testing and evaluation.

For non-Black adults, CKD diagnoses will likely decrease, especially among the elderly. One may anticipate confusion among reclassified individuals who had previously been told they had CKD, and so it will be helpful to develop best-practice approaches for patient education. Non-Black adults may see less reduction in drug doses because of higher estimated kidney function, and more liberal use of radiocontrast dyes in radiological studies. Fewer non-Black adults may be referred for dialysis preparation or kidney transplant consideration, although concomitantly the non-Black donor pool may potentially increase.

Because the overall prevalence of CKD stages 3-5 in the MHS is projected to decline by 18.8%, there is a possibility that this would be used to justify reallocation of strategic resources away from the CKD care. This would be an unsupported interpretation of data and an unintended and undesired consequence of the new equation, likely exacerbating rather than reducing disparities in the CKD outcomes.

In the active-duty population, the absolute prevalence of CKD was small, but the relative changes for Black versus non-Black adults with the 2021 equation were magnified when compared with the overall study population. In the military, even very early stages of CKD are of heightened concern because of deleterious effects on individual fitness-for-duty and retention and on unit readiness. As such, active-duty individuals with eGFR near 60 mL/min/1.73m^2^ are likely to undergo a more thorough diagnostic evaluation than normally seen in civilian practice.

The major strength of this study is that the MHS’s patient demographics, delivery systems, and quality of care parallel those found in private-sector health systems in the United States;^10^ thus, many of the findings are likely generalizable to the broader US population. Our real-world study population of nearly 2 million adults with 7 million serum creatinine values allows for more representative results, greater precision, and less bias. In addition, the data are longitudinal as opposed to one-time survey data, with rigorous chronicity criteria applied to the definition of CKD.

This study has several limitations. First, there was potential underestimation of CKD stages 3-5 prevalence by excluding individuals with only a single eGFR measurement of <60 mL/min/1.73m^2^ during the year. Our analysis showed that overall CKD prevalence increased 19% if those individuals were included. Second, as discussed, we did not include albuminuria in the criteria for CKD, which although having no effect on eGFR changes still leads to underestimation of overall CKD prevalence. Third, there were significant missing data on race in the nonactive-duty population, which we addressed using multiple imputations. Finally, any assessment of the future clinical effect of the revised CKD-EPI equation based on these data remains speculative, and there may be benefits and risks, which are currently unforeseen.

The removal of race as a factor in quantifying kidney function may be an important step in addressing disparities in the care and outcomes of chronic kidney disease. However, it is not without risk and the challenge is on health care providers and systems to maintain focus on both health and health care equity and personalized care in individuals with kidney disease. It might be important after implementation of the 2021 CKD-EPI equation to perform active surveillance of desired clinical outcomes and unintended consequences among different racial and ethnic groups. As recognized by the NKF-ASN Task Force,^6^ an ultimate goal is better assessments of eGFR by cystatin C and the development of next-generation race-independent markers of kidney function.
