# Supplementary material for: Impact of Race-Free Glomerular Filtration Rate Estimations on CKD Prevalence in the US Military Health System: A Retrospective Cohort Study
Source: Kidney Med. 2024 Jun 21;6(8):100861. doi: 10.1016/j.xkme.2024.100861 (PMC11295453; doi:10.1016/j.xkme.2024.100861)
Supplement: Supplementary File (PDF) — Figure S1; Table S1-S11. [file mmc1.docx]

Contents

[Supplementary Material 2](#_Toc162446284)

[Upper bound estimate for CKD crude prevalence 2](#_Toc162446285)

[Supplementary Table S1: Number of days between serum creatinine results used to define CKD Stages 3–5 strata. 3](#_Toc162446286)

[Supplementary Table S2: MHS demographics and eGFR by 2009 and 2021 CKD-EPI equations for all adults and races. 4](#_Toc162446287)

[Supplementary Table S2 a: complete case for all adults with ungrouped non-Black races. 5](#_Toc162446288)

[Supplementary Table S2 b: complete case for active-duty adults with ungrouped non-Black races. 6](#_Toc162446289)

[Supplementary Table S2 c: imputed race for all adults with grouped non-Black races. 7](#_Toc162446290)

[Supplementary Table S2 d: imputed race for active-duty adults with grouped non-Black races. 8](#_Toc162446291)

[Supplementary Table S2 e: imputed race for all adults with ungrouped non-Black races. 9](#_Toc162446292)

[Supplementary Table S2 f: imputed race for active-duty adults with ungrouped non-Black races. 10](#_Toc162446293)

[Supplementary Table S3: Upper bound estimate of CKD stages 3–5 prevalence. 11](#_Toc162446294)

[Supplementary Table S3 a: upper bound CKD stage 3–5 prevalence for complete case 11](#_Toc162446295)

[Supplementary Table S3 b: upper bound CKD stage 3–5 prevalence for imputed race 12](#_Toc162446296)

[Supplementary Figure S1: Mean absolute difference between eGFR values used to determine CKD stages 3–5 category as a function of the number of days between serum creatinine results. 13](#_Toc162446297)

Abbbreviations:

| AA/PI | Asian American and Pacific Islander |
| --- | --- |
| AI/AN | American Indian and Alaska Native |
| CI | Confidence interval |
| CKD | Chronic kidney disease |
| CKD-EPI | Chronic Kidney Disease Epidemiology Collaboration |
| eGFR | Estimated glomerular filtration range |
| IQR | Interquartile range |
| SD | Standard deviation |

# Supplementary Material

## Upper bound estimate for CKD crude prevalence

A total of 745,264 adults had only one creatinine measurement, and thus could not meet diagnostic criteria for CKD. In the complete case analysis, within this group the eGFR for the single measurement was < 60 mL/min/1.73m^2^ in 4,147 Black adults and 27,596 non-Black Adults using the 2009 equation and in 6,556 Black adults and 19,213 non-Black adults using the 2021 equation. With race imputation, the corresponding numbers were 1,273 (95% CI, 1198–1348) Black adults and 7,932 (95% CI, 7751–8114) non-Black adults using the 2009 equation and 2,477 (95%CI, 2344–2551) Black adults and 4,999 (95% CI, 4856–5142) non-Black adults using the 2021 equation.

By adding these “at-risk” adults to the CKD totals, we calculated an upper bound on the prevalence of CKD stages 3–5 as shown in Supplementary Tables S3a and S3b.

# Supplementary Table S1: Number of days between serum creatinine results used to define CKD Stages 3–5 strata.

| **Number of Days Between Labs** | **Number of Adults** | **Percent of Adults** | **Cumulative Percent of Adults** |
| --- | --- | --- | --- |
| **90–134** | **11,730** | **29.43** | **29.43** |
| **135–179** | **6,903** | **17.32** | **46.75** |
| **180–224** | **5,423** | **13.61** | **60.35** |
| **225–269** | **3,124** | **7.84** | **68.19** |
| **270–314** | **2,303** | **5.78** | **73.97** |
| **315–359** | **2,562** | **6.43** | **80.39** |
| **360–404** | **2,642** | **6.63** | **87.02** |
| **405–449** | **1,492** | **3.74** | **90.77** |
| **450–494** | **884** | **2.22** | **92.98** |
| **495–539** | **630** | **1.58** | **94.56** |
| **540–584** | **442** | **1.11** | **95.67** |
| **585–629** | **348** | **0.87** | **96.55** |
| **630–674** | **294** | **0.74** | **97.28** |
| **675–719** | **212** | **0.53** | **97.81** |
| **720–764** | **212** | **0.53** | **98.35** |
| **765–809** | **146** | **0.37** | **98.71** |
| **810–854** | **108** | **0.27** | **98.98** |
| **855–899** | **113** | **0.28** | **99.27** |
| **900–944** | **76** | **0.19** | **99.46** |
| **945–989** | **49** | **0.12** | **99.58** |
| **990–1034** | **32** | **0.08** | **99.66** |
| **1035–1079** | **36** | **0.09** | **99.75** |
| **1080–1124** | **24** | **0.06** | **99.81** |
| **1125–1169** | **29** | **0.07** | **99.88** |
| **1170–1214** | **15** | **0.04** | **99.92** |
| **1215–1259** | **14** | **0.04** | **99.96** |
| **1263–1426** | **17** | **0.04** | **100** |

For those adults determined to have CKD stages 3–5, the table shows the number of days between serum creatinine results with eGFR < 60 mL/min/1.73m^2^ used to make the determination. Calculations shown for the 2021 CKI-EPI equation.

# Supplementary Table S2: MHS demographics and eGFR by 2009 and 2021 CKD-EPI equations for all adults and races.

MHS demographics and CKD stages 3–5 number and prevalence by 2009 vs. 2021 CKD-EPI equations. For tables with race imputation, 95% confidence intervals are shown. Because the active-duty cohort had very few missing race (58 out of 754,169) and a low prevalence of CKD (0.11%), the CKD numbers were unaffected by race imputation. A confidence interval of (–) indicates the counts were unaffected by race imputation. Non-zero cell sizes less than 11 are not reported. *Not calculated due to cell size < 11 individuals.

|  | **All Adults** | | | | | | |
| --- | --- | --- | --- | --- | --- | --- | --- |
| **Race** | **All** | **AI/AN** | **AA/PI** | **Black** | **White** | **Other** | **Unknown** |
| **No.** | **1,502,607** | **14,112** | **87,571** | **282,890** | **865,023** | **53,889** | **199,122** |
| **Proportion, %** | **100.0** | **0.90** | **5.80** | **18.8** | **57.6** | **3.60** | **13.3** |
| **Female, n** | **532,445** | **4,929** | **32,111** | **107,373** | **219,890** | **18,540** | **149,602** |
| **Female proportion, %** | **35.4** | **34.9** | **36.7** | **38.0** | **25.4** | **34.4** | **75.1** |
| **Age, median (IQR), years** | **40 (28–54)** | **36 (28–47)** | **42 (32–54)** | **41 (29–54)** | **39 (28–53)** | **41 (30–54)** | **39 (26–52)** |
| **Active-duty, n** | **754,111** | **9,093** | **45,311** | **150,858** | **512,016** | **28,391** | **8,442** |
| **Active-duty proportion, %** | **50.2** | **64.4** | **51.7** | **53.3** | **59.2** | **52.7** | **4.24** |
| **Median 2009 eGFR (IQR) mL/min/1.73m2** | **91 (75–106)** | **96 (81–110)** | **92 (76–105)** | **95 (79–113)** | **91 (77–105)** | **92 (77–104)** | **81 (58–103)** |
| **Median 2021 eGFR (IQR) mL/min/1.73m2** | **93 (77–108)** | **100 (85–114)** | **97 (81–109)** | **87 (72–103)** | **96 (81–109)** | **97 (82–109)** | **87 (63–107)** |
| **eGFR change, median (IQR), mL/min/1.73m2** | **4 (2–5)** | **4 (3–5)** | **4 (3–5)** | **-8 (-11 to -6)** | **4 (3–5)** | **4 (3–5)** | **4 (3–5)** |
| **2009 CKD stages 3–5, n** | **31,743** | **156** | **1,567** | **4,147** | **11,610** | **749** | **13,514** |
| **2021 CKD stages 3–5, n** | **25,769** | **109** | **1,073** | **6,556** | **7,352** | **494** | **10,185** |
| **Change in CKD stages 3–5 n, %** | **-18.8** | **-30.1** | **-31.5** | **+58.1** | **-36.7** | **-34.0** | **-24.6** |
| **2009 CKD stages 3–5 crude prevalence, %** | **2.10** | **1.10** | **1.80** | **1.50** | **1.30** | **1.40** | **6.80** |
| **2021 CKD stages 3–5 crude prevalence, %** | **1.70** | **0.80** | **1.20** | **2.30** | **0.90** | **0.90** | **5.10** |
| **Change in crude prevalence, %** | **-0.40** | **-0.30** | **-0.60** | **+0.80** | **-0.40** | **-0.50** | **-1.70** |

## Supplementary Table S2 a: complete case for all adults with ungrouped non-Black races.

## Supplementary Table S2 b: complete case for active-duty adults with ungrouped non-Black races.

|  | **Active-duty Adults** | | | | | | |
| --- | --- | --- | --- | --- | --- | --- | --- |
| **Race** | **All** | **AI/AN** | **AA/PI** | **Black** | **White** | **Other** | **Unknown** |
| **No.** | **754,111** | **9,093** | **45,311** | **150,858** | **512,016** | **28,391** | **8,442** |
| **Proportion, %** | **100** | **1.20** | **6.00** | **20.0** | **67.9** | **3.80** | **1.10** |
| **Female, n** | **175,897** | **2,337** | **11,202** | **52,391** | **97,962** | **8,410** | **3,595** |
| **Female proportion, %** | **23.3** | **25.7** | **24.7** | **34.7** | **19.1** | **29.6** | **42.6** |
| **Age, median (IQR), years** | **31 (25–39)** | **31 (26–38)** | **34 (27–41)** | **31 (25–39)** | **31 (25–39)** | **32 (26–40)** | **27 (22–35)** |
| **Median 2009 eGFR (IQR) mL/min/1.73m2** | **101 (87–116)** | **103 (88–117)** | **99 (85–113)** | **105 (89–123)** | **100 (86–114)** | **100 (85–114)** | **105 (91–119)** |
| **Median 2021 eGFR (IQR) mL/min/1.73m2** | **102 (88–117)** | **106 (92–120)** | **103 (89–116)** | **94 (80–110)** | **104 (90–118)** | **103 (89–118)** | **108 (94–122)** |
| **eGFR change, median (IQR), mL/min/1.73m2** | **3 (0–4)** | **3 (3–4)** | **4 (3–4)** | **-11 (-13 to -9)** | **4 (3–4)** | **4 (3–4)** | **3 (3–4)** |
| **2009 CKD stages 3–5, n** | **838*** | **<11** | **72** | **216** | **512** | **28** | **<11** |
| **2021 CKD stages 3–5, n** | **858*** | **<11** | **41** | **523** | **274** | **16** | **<11** |
| **Change in CKD stages 3–5 n, %** | **+2.39** | ***** | **-43.1** | **+142** | **-46.5** | **-42.9** | ***** |
| **2009 CKD stages 3–5 crude prevalence, %** | **0.11** | ***** | **0.16** | **0.14** | **0.10** | **0.10** | ***** |
| **2021 CKD stages 3–5 crude prevalence, %** | **0.11** | ***** | **0.09** | **0.35** | **0.05** | **0.06** | ***** |
| **Change in crude prevalence, %** | **0.00** | ***** | **-0.07** | **+0.21** | **-0.05** | **-0.04** | ***** |

Non-zero cell sizes less than 11 are not reported. *sum includes unreported counts for AI/AN and Unknown races.

## Supplementary Table S2 c: imputed race for all adults with grouped non-Black races.

| **All Adults** | | | |
| --- | --- | --- | --- |
| **Race** | **All** | **Black** | **Non-Black** |
| **No. (95% CI)** | **1,970,433** | **355,314 (354,064–356,563)** | **1,615,119 (1,613,870–1,616,369)** |
| **Proportion (95% CI), %** | **100** | **18.0 (17.97–18.10)** | **82.0 (81.90–82.03)** |
| **Female (95% CI), n** | **968,374** | **176,235 (175,346–177,123)** | **792,139 (790,731–793,548)** |
| **Female proportion (95% CI), %** | **49.1** | **49.6 (49.4–49.8)** | **51.0 (50.9–51.0)** |
| **Age, median (IQR), years** | **40 (29–55)** | **44 (31–56)** | **40 (28–55)** |
| **Active-duty (95% CI), n** | **754,169** | **150,868 (150,291–151,446)** | **603,301 (602,096–604,506)** |
| **Active-duty proportion (95%CI), %** | **38.3** | **42.5 (42.3–42.6)** | **37.4 (37.4–37.4)** |
| **Median 2009 eGFR (IQR)**  **mL/min/1.73m^2^** | **91 (74–107)** | **95 (78–113)** | **90 (73–105)** |
| **Median 2021 eGFR (IQR)**  **mL/min/1.73m^2^** | **93 (76–108)** | **87 (72–102)** | **95 (78–109)** |
| **Median eGFR change (IQR), 2009 to 2021, mL/min/1.73m^2^** | **2 (2–5)** | **–8 (–11 to –6)** | **5 (3–5)** |
| **2009 CKD stages 3–5, n (95% CI)** | **48,954 (48,524–49,384)** | **5,828 (5669–5986)** | **43,126 (42,718–43,534)** |
| **2021 CKD stages 3–5, n (95% CI)** | **39,860 (–)** | **8,928 (8,735–9,121)** | **30,932 (30,586–31,279)** |
| **Change in CKD stages 3–5 n, %** | **–18.6** | **+53.2** | **–28.3** |
| **2009 CKD stages 3–5 crude prevalence, % (95% CI)** | **2.48 (2.46–2.51)** | **1.64 (1.60–1.68)** | **2.67 (2.65–2.70)** |
| **2021 CKD stages 3–5 crude prevalence, % (95% CI)** | **2.02 (–)** | **2.51 (2.46–2.57)** | **1.92 (1.89–1.94)** |
| **Change in crude prevalence, %** | **–0.46** | **+0.87** | **–0.75** |

## Supplementary Table S2 d: imputed race for active-duty adults with grouped non-Black races.

| **Active–Duty Adults** | | | |
| --- | --- | --- | --- |
| **Race** | **All** | **Black** | **Non–Black** |
| **No. (95% CI)** | **754,169** | **150,868 (150,186–151,549)** | **603,301 (602,620–603,982)** |
| **Proportion (95% CI), %** | **100** | **20.0 (19.9–20.1)** | **80.0 (79.9–80.1)** |
| **Female (95% CI), n** | **175,925** | **52,397 (52,034–52,759)** | **123,528 (122,914–124,143)** |
| **Female proportion (95% CI), %** | **23.3** | **34.7 (34.5–35.0)** | **20.5 (20.4–20.6)** |
| **Age, median (IQR), years** | **31 (25–39)** | **31 (35–39)** | **31 (25–39)** |
| **Median 2009 eGFR (IQR)**  **mL/min/1.73m^2^** | **101 (87–116)** | **105 (89–123)** | **100 (86–114)** |
| **Median 2021 eGFR (IQR)**  **mL/min/1.73m^2^** | **102 (88–117)** | **94 (80–110)** | **104 (90–118)** |
| **Median eGFR change (IQR), 2009 to 2021, mL/min/1.73m^2^** | **3 (0–4)** | **–11 (–13 to –9)** | **4 (3–4)** |
| **2009 CKD stages 3–5, n (95% CI)** | **838 (–)** | **216 (–)** | **622 (–)** |
| **2021 CKD stages 3–5, n (95% CI)** | **858 (–)** | **523 (–)** | **335 (–)** |
| **Change in CKD stages 3–5 n, %** | **+2.4** | **+142** | **–46.1** |
| **2009 CKD stages 3–5 crude prevalence, % (95% CI)** | **0.11 (–)** | **0.14 (0.12–0.16)** | **0.10 (0.10–0.11)** |
| **2021 CKD stages 3–5 crude prevalence, % (95% CI)** | **0.11 (–)** | **0.35 (0.32–0.38)** | **0.06 (0.05–0.06)** |
| **Change in crude prevalence, %** | **0** | **+0.20** | **–0.05** |

Because the active-duty cohort had very few missing race (58 out of 754,169) and a low prevalence of CKD (0.11%), the CKD numbers were unaffected by race imputation. A confidence interval of (–) indicates the counts were unaffected by race imputation.

|  | **All Adults** | | | | | | |
| --- | --- | --- | --- | --- | --- | --- | --- |
| **Race** | **All** | **AI/AN** | **AA/PI** | **Black** | **White** | **Other** | **Unknown** |
| **No. (95% CI)** | **1,970,433** | **16,943 (16,676–17,210)** | **110,990 (110,242–111,739)** | **355,314 (354,064–356,563)** | **1,016,973 (1,015,289–1,018,657)** | **67,324 (66,724–67,923)** | **402,889 (401,481–404,298)** |
| **Proportion (95% CI), %** | **100** | **0.86 (0.85–0.87)** | **5.63 (5.59–5.67)** | **18.0 (17.97–18.10)** | **51.6 (51.5–51.7)** | **3.42 (3.39–3.45)** | **20.5 (20.4–20.5)** |
| **Female (95% CI), n** | **968,374** | **7,549 (7,400–7,698)** | **54,327 (53,799–54,854)** | **176,235 (175,346–177,123)** | **353,823 (352,482–355,164)** | **31,087 (30,672–31,502)** | **345,353 (344,380–346,327)** |
| **Female proportion (95% CI), %** | **49.1** | **44.6 (43.8–45.4)** | **48.9 (48.6–49.3)** | **49.6 (49.4–49.8)** | **34.8 (34.7–34.9)** | **46.2 (45.7–46.6)** | **85.7 (85.6–85.8)** |
| **Age, median (IQR), years** | **40 (29–55)** | **37 (29–49)** | **44 (33–55)** | **44 (31–56)** | **40 (28–54)** | **43 (31–56)** | **37 (26–62)** |
| **Active-duty (95% CI), n** | **754,169** | **9,094 (8,967–9,221)** | **45,315 (44,994–45,636)** | **150,868 (150,291–151,446)** | **512,055 (511,066–513,043)** | **28,393 (28,142–28,645)** | **8,444 (8,266–8,622)** |
| **Active-duty proportion (95%CI), %** | **38.3** | **53.7 (52.88–54.47)** | **40.8 (40.50–41.15)** | **42.5 (42.3–42.6)** | **50.4 (50.2–50.5)** | **42.2 (41.75–42.60)** | **2.1 (2.1–2.1)** |
| **Median 2009 eGFR (IQR)**  **mL/min/1.73m^2^** | **91 (74–107)** | **96 (80–110)** | **92 (77–105)** | **95.0 (78.0–113)** | **92 (77–105)** | **92 (77–104)** | **84 (59–106)** |
| **Median 2021 eGFR (IQR)**  **mL/min/1.73m^2^** | **93 (76–108)** | **100 (85–114)** | **97 (82–109)** | **87.0 (72.0–102)** | **97 (82–109)** | **97 (82–108)** | **89 (65–109)** |
| **eGFR change, median (IQR), mL/min/1.73m2** | **4 (2–5)** | **4 (3–5)** | **4 (3–5)** | **–8.00 (–11.00 – –6.00)** | **4 (3–5)** | **4 (3–5)** | **4 (3–5)** |
| **2009 CKD stages 3–5, n (95% CI)** | **48,954** | **192 (162–223)** | **1,913 (1,818–2,008)** | **5,828 (5,669–5,986)** | **13,645 (13,406–13,885)** | **955 (887–1,024)** | **26,421 (26,104–26,737)** |
| **2021 CKD stages 3–5, n (95% CI)** | **39,860** | **135 (110–160)** | **1,307 (1,228–1,386)** | **8,928 (8,735–9,121)** | **8,597 (8,525–8,790)** | **626 (571–680)** | **20,268 (19,989–20,547)** |
| **Change in CKD stages 3–5 n, %** | **-18.6** | **-30.0** | **-31.7** | **+53.2** | **-37.0** | **-34.5** | **-23.3** |
| **2009 CKD stages 3–5 crude prevalence, % (95% CI)** | **2.48 (2.46–2.51)** | **1.14 (0.96–1.31)** | **1.72 (1.64–1.81)** | **1.64 (1.60–1.68)** | **1.34 (1.32–1.34)** | **1.42 (1.32–1.52)** | **6.56 (6.48–6.4)** |
| **2021 CKD stages 3–5 crude prevalence, % (95% CI)** | **2.02** | **0.79 (0.65–0.94)** | **1.18 (1.11–1.25)** | **2.51 (2.46–2.57)** | **0.85 (0.83–0.85)** | **0.93 (0.85–1.01)** | **5.03 (4.96–5.1)** |
| **Change in crude prevalence, %** | **-0.46** | **-0.34** | **-0.55** | **+0.87** | **-0.50** | **-0.49** | **-1.53** |

## Supplementary Table S2 e: imputed race for all adults with ungrouped non-Black races.

## Supplementary Table S2 f: imputed race for active-duty adults with ungrouped non-Black races.

|  | **Active-Duty Adults** | | | | | | |
| --- | --- | --- | --- | --- | --- | --- | --- |
| **Race** | **All** | **AI/AN** | **AA/PI** | **Black** | **White** | **Other** | **Unknown** |
| **No. (95% CI)** | **754,169** | **9,094 (8,908–9,280)** | **45,315 (44,910–45,719)** | **150,868 (150,186–151,549)** | **512,055 (511,260–512,849)** | **28,393 (28,069–28,717)** | **8,444 (8,265–8,623)** |
| **Proportion (95% CI), %** | **100** | **1.21 (1.18–1.23)** | **6.01 (5.95–6.06)** | **20.0 (19.9–20.1)** | **67.9 (67.8–68.0)** | **3.76 (3.72–3.81)** | **1.1 (1.1–1.1)** |
| **Female (95% CI), n** | **175,925** | **2,338 (2,256–2,419)** | **11,204 (11,024–11,384)** | **52,397 (52,034–52,759)** | **97,978 (97,426–98,530)** | **8,411 (8,261–8,562)** | **3,597 (3,508–3,686)** |
| **Female proportion (95% CI), %** | **23.3** | **25.7 (24.8–26.6)** | **24.7 (24.3–25.1)** | **34.7 (34.5–35.0)** | **19.1 (19.0–19.2)** | **29.6 (29.1–30.2)** | **42.6 (41.5–43.7)** |
| **Age, median (IQR), years** | **31 (25–39)** | **31 (26–38)** | **34 (27–41)** | **31.0 (35.0–39.0)** | **31 (25–39)** | **32 (26–40)** | **27 (22–35)** |
| **Median 2009 eGFR (IQR)**  **mL/min/1.73m^2^** | **101 (87–116)** | **103 (88–117)** | **99 (85–113)** | **105 (89.0–123)** | **100 (86–114)** | **100 (85–114)** | **105 (91–119)** |
| **Median 2021 eGFR (IQR)**  **mL/min/1.73m^2^** | **102 (88–117)** | **106 (92–120)** | **103 (89–116)** | **94 (80.0–110)** | **104 (90–118)** | **103 (89–117)** | **108 (94–122)** |
| **eGFR change, median (IQR), mL/min/1.73m2** | **3 (0–4)** | **3 (3–4)** | **4 (3–4)** | **–11.00 (–13.00 to –9.00)** | **4 (3–4)** | **4 (3–4)** | **3 (3–4)** |
| **2009 CKD stages 3–5, n (95% CI)** | **838** | **<11 (–)** | **72 (–)** | **216 (–)** | **512 (–)** | **28 (–)** | **<11 (–)** |
| **2021 CKD stages 3–5, n (95% CI)** | **858** | **<11 (–)** | **41 (–)** | **523 (–)** | **274 (–)** | **16 (–)** | **<11 (–)** |
| **Change in CKD stages 3–5 n, %** | **+2.39** | ***** | **–43.1** | **+142** | **–46.5** | **–42.9** | ***** |
| **2009 CKD stages 3–5 crude prevalence, % (95% CI)** | **0.11 (–)** | *** (–)** | **0.16 (–)** | **0.14% (0.12–0.16)** | **0.10 (0.1–0.1)** | **0.10 (–)** | *** (–)** |
| **2021 CKD stages 3–5 crude prevalence, % (95% CI)** | **0.11** | *** (–)** | **0.09 (–)** | **0.35 (0.32–0.38)** | **0.05 (–)** | **0.06 (–)** | ***(–)** |
| **Change in crude prevalence, %** | **0** | ***** | **-0.07** | **0.20** | **-0.05** | **-0.04** | ***** |

Because the active-duty cohort had very few missing race (58 out of 754,169) and a low prevalence of CKD (0.11%), the CKD numbers were unaffected by race imputation. A confidence interval of (–) indicates the counts were unaffected by race imputation.

# Supplementary Table S3: Upper bound estimate of CKD stages 3–5 prevalence.

## Supplementary Table S3 a: upper bound CKD stage 3–5 prevalence for complete case

|  | **CKD stage 3–5 n** | **“at risk” for CKD: single eGFR of < 60 mL/min/1.73m2 n** | **Upper estimate =  (CKD stage 3–5) + (“at risk”) n** | **Change in n** | **CKD stage 3–5 crude prevalence, %** | **Upper estimate**  **CKD stage 3–5 crude prevalence, %** | **Change in crude prevalence %** |
| --- | --- | --- | --- | --- | --- | --- | --- |
| **All Adults 2009 CKD-EPI** | **31,743** | **6,716** | **38,459** | **+21.2** | **2.11** | **2.56** | **+0.45** |
| **Black Adults 2009 CKD-EPI** | **4,147** | **838** | **4,985** | **+20.2** | **1.47** | **1.76** | **+0.29** |
| **Non-Black Adults 2009 CKD-EPI** | **27,596** | **5,878** | **33,474** | **+21.3** | **2.26** | **2.74** | **+0.48** |
| **All Adults 2021 CKD-EPI** | **25,769** | **5,311** | **31,080** | **+20.6** | **1.71** | **2.07** | **+0.36** |
| **Black Adults 2021 CKD-EPI** | **6,556** | **1,729** | **8,285** | **+26.4** | **2.32** | **2.93** | **+0.61** |
| **Non-Black Adults 2021 CKD-EPI** | **19,213** | **3,582** | **22,795** | **+18.6** | **1.58** | **1.87** | **+0.29** |

## Supplementary Table S3 b: upper bound CKD stage 3–5 prevalence for imputed race

|  | **CKD stage 3–5**  **n (95% CI)** | **“at risk” for CKD: single eGFR of < 60 mL/min/1.73m^2^**  **n (95% CI)** | **Upper estimate =**  **(CKD stage 3–5) + (“at risk”)**  **n (95% CI)** | **Change in n** | **CKD stage 3–5 crude prevalence,**  **% (95% CI)** | **Upper estimate of CKD 3–5 crude prevalence,**  **% (95% CI)** | **Change in crude prevalence %** |
| --- | --- | --- | --- | --- | --- | --- | --- |
| **All Adults**  **2009 CKD-EPI** | **48,954 (48,524–49,384)** | **9,205 (9,014–9,396)** | **58,159 (57,690–58,629)** | **+18.8** | **2.48 (2.46–2.51)** | **2.95 (2.93–2.98)** | **+0.47** |
| **Black Adults**  **2009 CKD-EPI** | **5,828 (5,669–5,986)** | **1,273 (1,198–1,348)** | **7,100 (6,925–7,276)** | **+21.8** | **1.64 (1.60–1.68)** | **2.00 (1.95–2.05)** | **+0.36** |
| **Non-Black Adults**  **2009 CKD-EPI** | **43,126 (42,718–43,534)** | **7,932 (7,751–8,114)** | **51,059 (50,613–51,505)** | **+18.4** | **2.67 (2.65–2.70)** | **3.16 (3.13–3.18)** | **+0.49** |
| **All Adults**  **2021 CKD-EPI** | **39,860 (–)** | **7,446 (–)** | **47,306 (–)** | **+18.7** | **2.02 (–)** | **2.4 (–)** | **+0.38** |
| **Black Adults**  **2021 CKD-EPI** | **8,928 (8,735–9,121)** | **2,447 (2,344–2,551)** | **11,375 (11,155–11,594)** | **+27.4** | **2.51 (2.46–2.57)** | **3.20 (3.14–3.26)** | **+0.69** |
| **Non-Black Adults**  **2021 CKD-EPI** | **30,932 (30,586–31,279)** | **4,999 (4,856–5,142)** | **35,931 (35,556–36,306)** | **+16.2** | **1.92 (1.89–1.94)** | **2.22 (2.20–2.25)** | **+0.31** |

# Supplementary Figure S1: Mean absolute difference between eGFR values used to determine CKD stages 3–5 category as a function of the number of days between serum creatinine results.

Mean absolute difference |Δ eGFR| between eGFR values used to determine CKD stages 3–5 category as a function of the number of days between serum creatinine results. The mean absolute difference (±SD) across all intervals was 5.3±5.1 mL/min/1.73m^2^. Error bars represent ±1 standard deviation.

Abbreviations: CKD, chronic kidney disease; eGFR, estimated glomerular filtration rate; SD, standard deviation
